# Draft Genome Sequences of *Anaerolinea thermolimosa* IMO-1, *Bellilinea caldifistulae* GOMI-1, *Leptolinea tardivitalis* YMTK-2, *Levilinea saccharolytica* KIBI-1, *Longilinea arvoryzae* KOME-1, Previously Described as Members of the Class *Anaerolineae* (*Chloroflexi*)

**DOI:** 10.1128/genomeA.00975-15

**Published:** 2015-09-17

**Authors:** Norihisa Matsuura, Dieter M. Tourlousse, Akiko Ohashi, Philip Hugenholtz, Yuji Sekiguchi

**Affiliations:** a Biomedical Research Institute, National Institute of Advanced Industrial Science and Technology (AIST), Tsukuba, Ibaraki, Japan; b Australian Centre for Ecogenomics, School of Chemistry and Molecular Biosciences, The University of Queensland, St. Lucia, Queensland, Australia; c Institute for Molecular Bioscience, The University of Queensland, St. Lucia, Queensland, Australia

## Abstract

Members of the class *Anaerolineae* in the bacterial phylum *Chloroflexi* are widespread in a range of ecosystems but remain poorly understood. We present here the draft genome sequences of the type strains of five *Anaerolineae* species, *Anaerolinea thermolimosa* IMO-1, *Bellilinea caldifistulae* GOMI-1, *Leptolinea tardivitalis* YMTK-2, *Levilinea saccharolytica* KIBI-1, and *Longilinea arvoryzae* KOME-1.

## GENOME ANNOUNCEMENT

Members of the class *Anaerolineae* are cosmopolitan bacteria found in various ecosystems, suggesting their functional importance (1). Currently, 10 *Anaerolineae* species have been isolated and characterized (2–8). However, the genome sequence of only a single species, *Anaerolinea thermophila* (strain UNI-1), has been available. To further expand the knowledge on the genomic diversity of the *Anaerolineae*, we generated draft genomes of five previously described isolates. Two represent the type strains of thermophilic species, *Anaerolinea thermolimosa* (strain IMO-1) and *Bellilinea caldifistulae* (strain GOMI-1), isolated from thermophilic anaerobic waste/wastewater treatment systems (3, 4). The others are the type strains of mesophilic species, *Leptolinea tardivitalis* (strain YMTK-2), *Levilinea saccharolytica* (strain KIBI-1), and *Longilinea arvoryzae* (strain KOME-1), isolated from mesophilic anaerobic wastewater treatment systems or Japanese paddy field soil (3, 4).

Nextera XT paired-end (300 to 700 bp) and Nextera mate-pair (2 to 10 kbp) libraries were prepared from the genomic DNA of each strain. Pooled libraries were sequenced on an Illumina MiSeq (2 × 250-bp reads) at an expected coverage of >50× and >10× per genome for the paired-end and mate-pair libraries, respectively. The sequence reads were merged with SeqPrep with concurrent removal of sequencing adapters. Unmerged reads were quality trimmed and filtered using Nesoni version 0.112. SPAdes version 2.5.0 (9) was used for assembly, and further scaffolding and refinement were performed as described previously (10). Genome annotations were generated within the Integrated Microbial Genomes platform (11).

The assembly of the *A. thermolimosa* IMO-1 genome consists of 81 contigs in 6 scaffolds (coverage, 180×); the total size is 4,173,865 bp, and the G+C content is 53.72%. The assembly of the *B. caldifistulae* GOMI-1 genome consists of 43 contigs in a single scaffold (coverage, 180×); the total size is 3,698,317 bp, and the G+C content is 52.18%. The assembly of the *L. tardivitalis* YMTK-2 genome consists of 15 contigs in 15 scaffolds (coverage, 230×); the total size is 3,687,036 bp, and the G+C content is 46.82%. The assembly of the *L. saccharolytica* KIBI-1 genome consists of 196 contigs in 4 scaffolds (coverage, 200×); the total size is 4,249,622 bp, and the G+C content is 57.36%. The assembly of the *L. arvoryzae* KOME-1 genome consists of 25 contigs in 2 scaffolds (coverage, 60×); the total size is 4,438,311 bp, and the G+C content is 56.84%. The genomes are predicted to contain between 3,301 (*L. tardivitalis* YMTK-2) and 3,888 (*L. arvoryzae* KOME-1) protein-coding genes and between 54 (*L. tardivitalis* YMTK-2) and 64 (*A. thermolimosa* IMO-1) RNAs. The availability of these genomes will contribute to our understanding of the metabolic potential and possible ecological roles of members of the class *Anaerolineae*.

### Nucleotide sequence accession numbers.

These whole-genome shotgun projects have been deposited at DDBJ/EMBL/GenBank under the accession numbers BBXW00000000, BBXX00000000, BBXY00000000, BBXZ00000000, and BBYA00000000 for *A. thermolimosa* IMO-1 JCM 12577, *B. caldifistulae* GOMI-1 JCM 13669, *L. arvoryzae* KOME-1 JCM 13670, *L. saccharolytica* KIBI-1 JCM 12578, and *L. tardivitalis* YMTK-2 JCM 12579, respectively. The versions described in this paper are versions BBXW01000000, BBXX01000000, BBXY01000000, BBXZ01000000, and BBYA01000000.
